# Toward high-quality bowel preparation in Italy: insights from a nationwide cross-sectional survey of endoscopists

**DOI:** 10.1177/26317745261441726

**Published:** 2026-05-11

**Authors:** Cesare Hassan, Roberto Di Mitri, Lorenzo Fuccio, Marcello Maida, Gianpiero Manes, Mauro Manno, Franco Radaelli, Cristiano Spada, Roberto Vassallo, Alessandro Repici

**Affiliations:** IRCCS Humanitas Research Hospital, Department of Gastroenterology, via Manzoni Rozzano, Milano, Italy; Department of Biomedical Sciences, Humanitas University, via Rita Levi Montalcini, Pieve Emanuele, Milano, Italy; UOC Gastroenterologia con Endoscopia Digestiva ARNAS Ospedali “Civico - Di Cristina - Benfratelli,” Palermo, Italy; Department of Medical Sciences and Surgery, University of Bologna, Bologna, Italy; Gastroenterology Unit, RCCS-Azienda Ospedaliero-Universitaria di Bologna, Bologna, Italy; Department of Medicine and Surgery, University of Enna ‘Kore’, Enna, Italy; Gastroenterology Unit, Umberto I Hospital, Enna, Italy; Department of Gastroenterology, ASST Rhodense, Garbagnate Milanese and Rho Hospitals, Milano, Italy; Gastroenterology and Digestive Endoscopy Unit, Azienda USL Modena, Carpi Hospital, Carpi, Italy; Gastroenterology Unit, Valduce Hospital, Como, Italy; Digestive Endoscopy Unit, Fondazione Policlinico Universitario Agostino Gemelli IRCCS, Rome, Italy; Poliambulanza Foundation Hospital Institute, Brescia, Italy; UOS Gastroenterologia, Ospedale Buccheri la Ferla Fatebenefratelli, Palermo, Italy; IRCCS Humanitas Research Hospital, Department of Gastroenterology, Milano, Italy; Department of Biomedical Sciences, Humanitas University, via Rita Levi Montalcini, Pieve Emanuele, Milano, Italy

**Keywords:** adenoma detection rate, bowel preparation, colonoscopy, colorectal cancer screening, quality colonoscopy

## Abstract

**Background:**

Understanding endoscopists’ perspectives and routine practice offers opportunities to improve bowel cleansing for colonoscopy.

**Objective:**

To elucidate Italian endoscopists’ perceptions of bowel preparation quality, focusing on defining high-quality cleansing (HQC) and its perceived benefits in clinical practice and for diagnostic outcomes.

**Design:**

Nationwide, cross-sectional, web-based survey.

**Methods:**

A nationwide, web-based cross-sectional survey was undertaken in Italy between August and September 2024 among gastroenterologists with special interest in endoscopy. Participants were recruited via telephone screening; of 498 gastroenterologists contacted, 150 respondents completed an online questionnaire; analyses were descriptive.

**Results:**

The survey results revealed that all respondents (100%) routinely evaluate and document cleansing in the endoscopy report and almost all (99%) used validated scales. The majority (72%) of endoscopists aimed for HQC, which they defined as a segment score of ⩾8–9 on the Boston Bowel Preparation Scale or ‘excellent’ on the Aronchick scale. Almost all (93%) considered HQC important in every colonoscopy regardless of indication. All respondents considered that HQC allows higher identification rates for adenomas and sessile serrated lesions, reduces procedure time, and improves overall clinical efficiency; 99% considered that HQC allows for more appropriate surveillance intervals. On a scale of 1–10 to rate confidence with the diagnostic reliability of the exam (1 = not at all confident, 10 = very confident), the respondents’ levels of confidence improved with high-quality bowel preparation; mean scores were 2.1 with inadequate preparation, 6.6 with good cleansing and 9.2 with high-quality bowel cleansing.

**Conclusion:**

The survey revealed that the vast majority of Italian endoscopists consider HQC essential across all clinical indications. The results support the transition from ‘good’ to ‘high-quality’ cleansing as the new standard in clinical colonoscopy practice.

## Plain language summary

High-quality bowel preparation in Italy

Understanding endoscopists’ perspectives and routine practice offers opportunities to improve bowel cleansing for colonoscopy. A survey of Italian endoscopists was conducted to elucidate perceptions of bowel preparation quality, focusing on defining high-quality cleansing (HQC) and its benefits in clinical practice and for diagnostic outcomes. The survey results revealed that all respondents (100%) routinely evaluate and document cleansing in the endoscopy report and almost all (99%) used validated scales. The majority (72%) of endoscopists aimed for HQC, which they defined as a segment score of ⩾8–9 on the Boston Bowel Preparation Scale or “excellent” on the Aronchick scale. Almost all (93%) considered HQC important in every colonoscopy regardless of indication. All respondents considered that HQC allows higher identification rates for adenomas and sessile serrated lesions, reduces procedure time, and improves overall clinical efficiency; 99% considered that HQC allows for more appropriate surveillance intervals. On a scale of 1 to 10 to rate confidence with the diagnostic reliability of the exam (1 = not at all confident, 10 = very confident), the respondents’ levels of confidence improved with high-quality bowel preparation; mean scores were 2.1 with inadequate preparation, 6.6 with good cleansing, and 9.2 with high-quality bowel cleansing. The survey revealed that the vast majority of Italian endoscopists consider HQC essential across all clinical indications. The results support the transition from “good” to “high-quality” cleansing as the new standard in clinical colonoscopy practice.

## Introduction

Colonoscopy plays a pivotal role in the prevention of colorectal cancer (CRC) and significantly decreases CRC incidence and mortality.^1,2^ However, its success is highly dependent on the quality of bowel preparation. Bowel preparation quality is associated with two key performance measures for colonoscopy, namely adenoma detection rate (ADR) and caecal intubation rate.^3^ Inadequate or suboptimal bowel preparation leads to unnecessary costs and patient inconvenience as the examination has to be repeated or the subsequent surveillance examination has to be rescheduled at a shorter-than-usual interval.^4–7^

To ensure validity and reliability of bowel preparation quality, clinical guidelines recommend documenting the adequacy of the preparation using validated scales,^5,8^ including the Boston Bowel Preparation Scale (BBPS),^9^ the Aronchick scale,^10,11^ the Ottawa Bowel Preparation Scale,^12^ the Harefield Cleansing Scale^13^ and the Chicago Bowel Preparation Scale.^14^ These scales are inherently subjective and are heavily influenced by the operator’s preferences and experience.^15–17^ The BBPS and the Aronchick scale are both commonly used in clinical practice, whereas the other scales have been mainly adopted in clinical trials;^15,18^ current guidelines recommend the BBPS as the preferred scale.^7,19^ The BBPS is the most extensively validated bowel cleansing score, with a score of ⩾2 in each colon segment correlating with adequate preparation.^20^ Patients with adequate bowel preparation scores should have intervals for the next screening or surveillance examination that are consistent with current screening or post-polypectomy surveillance guidelines. United States (US)-based^8^ and European-based^5^ guidelines have recommended that patients with inadequate preparation should have their examination repeated within 1 year.^5,8^

There is ongoing debate about whether high scores of bowel preparation indicating ‘high-quality’ rather than merely ‘good’ preparation are associated with improved colonoscopy outcomes. There is also no clear consensus on how to define high-quality cleansing (HQC).^21^ Various researchers have examined the impact of HQC on colonoscopy outcomes.^3,10,22^ HQC ensures optimal mucosal visualisation, minimises the risk of missing potentially malignant lesions^4,23–25^ and reduces the cost burden associated with procedures repeated at shorter intervals.^4–6^ In 2016, a clinical study demonstrated that HQC is significantly more effective than intermediate-quality cleansing in detecting sessile serrated polyps (SSPs).^26^ Similarly, in 2020, a study confirmed a significantly higher ADR was obtained with HQC than with good preparation.^27^ In a large population-based screening programme database in Austria, compared with HQC, the probability of adenoma detection was significantly reduced with fair rather than poor and inadequate- bowel preparation at screening colonoscopy.^17^ Fair bowel preparation also significantly increased the risk of mortality from post-colonoscopy CRC (PCCRC) compared with HQC.^17^ Despite methodological differences in these three studies, they all highlight the importance – if not the necessity – of achieving high-quality preparation.^28^

Since these findings suggest that HQC is probably better than adequate bowel preparation for screening colonoscopies, this leads to the central question of what practising gastroenterologists/endoscopists do in the real world. Understanding endoscopists’ perspectives on HQC offers valuable insights into current challenges and opportunities to improve bowel cleansing for colonoscopy. To investigate this further, an exploratory, nationwide, cross-sectional survey was conducted among Italian endoscopists to assess perceptions of bowel preparation quality, focusing on defining HQC and identifying its perceived benefits in clinical practice and for diagnostic outcomes.

## Methods

### Survey questionnaire and advisory board meeting

An exploratory, cross-sectional, web-based survey (online questionnaire) was conducted to describe endoscopists’ self-reported practices and perceptions regarding bowel preparation quality. The nationwide survey was carried out in Italy between 27 August and 26 September 2024 among gastroenterologists with a special interest in endoscopy and was administered by Ipsos (Milan, Italy), a research agency specialising in healthcare and social research. Survey questions were developed with the help of a Key Opinion Leader in colonoscopy and finalised by Ipsos. The survey responses were compiled and analysed at a meeting held on 12 December 2024 that involved 10 gastroenterologists with special interest in endoscopy from leading Italian hospitals and outpatient clinics (the authors), all of whom are experts in their fields and have amassed many years of experience in assessing bowel preparation quality for colonoscopies based on both clinical and research.

### Selection of endoscopists

Gastroenterologists with endoscopic expertise were recruited by telephone by interviewers from Ipsos trained in health surveys who verified eligibility against predefined screening criteria. Eligible participants were aged ⩽65 years, had ⩾5 years’ experience performing colonoscopies, worked predominantly in public hospitals, accredited private hospitals or public outpatient clinics (excluding those practising predominantly in private practice), and performed ⩾200 colonoscopies per year. To reflect routine clinical practice and limit selection bias towards academically visible early adopters, we excluded endoscopists classified as ‘opinion leaders’, operationally defined as those who had served as speakers at national/international colonoscopy conferences and/or had authored colonoscopy-related publications within the previous 2 years. We also excluded physicians not meeting the minimum volume and setting criteria described above. Of the 498 gastroenterologists contacted by telephone, 150 met the eligibility criteria and completed the survey; 328 did not meet screening criteria and 20 declined participation. Given the self-reported nature of survey data, potential selection and response biases are addressed in the Limitations section.

### Survey data collection

The survey consisted of a structured questionnaire comprising 11 closed-ended questions and one 10-point Likert scale item (Supplemental Material). The survey was structured into two sections: section (1) had questions pertaining to the endoscopist’s demographic and professional background, the clinical setting, the number of procedures conducted and their routine practice; and section (2) had questions related to the endoscopist’s evaluation of, and response to, bowel preparation, and how they assess, define and emotionally react to varying levels of bowel cleansing quality. Finally, the survey respondents rated their sense of confidence with the diagnostic reliability of the exam with varying bowel cleansing qualities, using a Likert scale ranging from 1 (not at all confident) to 10 (very confident) based on the quality of bowel preparation. No objective validation (e.g. audit of endoscopy reports) was performed.

### Statistical analysis

Statistical analysis was descriptive. Categorical variables were summarised as frequencies and percentages, and continuous variables as means. No inferential tests or multivariable adjustments were performed, given the exploratory nature of the study.

The reporting of this study conforms to the Strengthening the Reporting of Observational Studies in Epidemiology (STROBE) statement for cross-sectional studies.^29^

## Results

### Survey respondents

The 150 respondents had a mean age of 53.4 years and 21.8 years of professional experience, primarily in public hospitals (76%), with most respondents based in the northwestern (31%) or southern (32%) regions of Italy (Table 1).

**Table 1.** Demographic and clinical practice characteristics of the Italian physicians who completed the survey.^a^.

Variable	*N* = 150
Age, years	
⩽45	24%
46–55	22%
56–60	22%
61–65	26%
Mean	53.4
Years in profession	
5–15	27%
16–20	19%
21–25	18%
>25	36%
Mean	21.8
Geographical area of Italy	
Northwest	31%
Northeast	17%
Centre	20%
South and Islands	32%
Primary workplace	
Public hospital	76%
Private accredited hospital	22%
Private hospital	1%
Local Health Authority clinic	1%
Number of colonoscopies performed per year	
200–499	30%
500–549	20%
550–599	27%
⩾600	23%
Mean	589

Data are presented as percentage of physicians, unless specified otherwise.

a Eligible participants were gastroenterologists with endoscopic expertise, aged ⩽65 years, with ⩾5 years of experience performing colonoscopies, working in public hospitals, accredited private hospitals, or public outpatient clinics, and performing ⩾200 colonoscopies per year.

### Desired quality of bowel preparation

Most physicians (86%) reported that they aim for HQC in their routine clinical practice. Very few reported that they targeted a good level of cleansing (8%) or one sufficient to perform the examination (6%). Almost all respondents (93%) considered HQC important in every colonoscopy, regardless of indication; the remaining 7% deemed it essential only for selected indications, such as faecal immunochemical test (FIT)-positive CRC screening programmes (Table 2).

**Table 2.** Evaluation, definition and response to bowel preparation reported by the Italian physicians who completed the survey.

Variable	*N* = 150
Use of validated scales	
Yes	99%
No	1%
Aim of bowel preparation	
Optimal (high-quality) cleansing	86%
Good cleansing	8%
Sufficient cleansing to address the clinical question	6%
Definition of adequate bowel preparation	
Meets validated criteria in bowel preparation quality scales (BBPS score ⩾2; ‘fair’ rating on the Aronchick scale)	83%
Ensures clear mucosal visibility with minimal washing and suctioning	13%
A cleansing quality that allows confident diagnosis	4%
Definition of high-quality bowel preparation	
High scores (BBPS 8–9; ‘excellent’ rating on the Aronchick scale)	81%
Optimal visualisation without washing and suctioning	10%
Supports a confident diagnosis by the endoscopist	9%
Benefits of high-quality cleansing	
Higher adenoma detection rate	100%
Higher sessile serrated lesion detection rate	100%
Reduced examination time and improved efficiency	100%
Better appropriateness of surveillance intervals	99%
Importance of optimal bowel preparation	
In every colonoscopy	93%
Mainly for certain indications (e.g. a positive FIT result for screening colonoscopy)	7%

Data are reported as percentage of patients.

BBPS, Boston Bowel Preparation Scale; FIT, faecal immunochemical test.

### Assessment and definition of bowel preparation quality

All respondents (100%) reported routinely evaluating and documenting bowel cleansing in the endoscopy report; almost all (99%) reported that they use validated bowel preparation quality scales (Table 2).

Most (83%) endoscopists defined good cleansing according to definitions of validated scales, in particular as a segment score of ⩾2 on the BBPS or an ‘at least fair’ on the Aronchick rating.^9,11^ A further 13% defined it as mucosa visibility without excessive washing or suction, and 4% defined it as providing sufficient clarity to make a reliable diagnosis. HQC was defined by 81% of endoscopists as BBPS scores of 8–9 or ‘excellent’ on the Aronchick scale.^9,11^ Ten per cent associated HQC with optimal visualisation of the mucosa without needing washing and suctioning, while 9% defined it as enabling a confident diagnosis by the endoscopist.

### Benefits of high-quality bowel preparation

The physicians agreed that HQC is associated with several benefits including higher ADR and SSL detection rates (100% of respondents, respectively), more appropriate surveillance intervals (99%), reduced examination time (100%) and enhanced endoscopy efficiency (100%).

**Figure 1.**
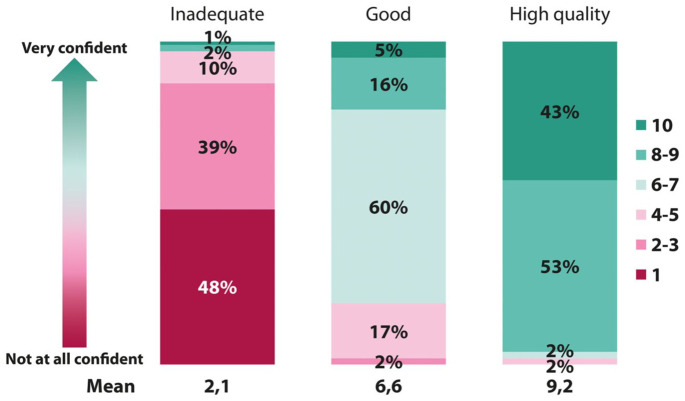
Confidence scores among endoscopists after performing colonoscopies with different bowel cleansing qualities. Confidence scores were rated on a 10-point scale, where 1 = not at all confident and 10 = very confident, based on the quality of bowel preparation.

Confidence with the diagnostic reliability of the exam was higher with HQC than with good cleansing (mean score of 9.2 vs 6.6) and was lowest with inadequate preparation (mean score of 2.1; Figure 1).

## Discussion

The survey findings indicate that assessment of bowel preparation quality is a systematic practice amongst the Italian endoscopists surveyed. This finding agrees with the results of another recent nationwide Italian survey, which reported that 93.9% of participating endoscopists stated that they used the BBPS for assessing bowel preparation quality.^30^ This is an improvement over colonoscopy practice in Italy from around 20 years ago, when a survey found that only 108 (39.0%) of the 278 participating endoscopy centres were systematically reporting the quality of bowel cleansing.^31^

The majority of endoscopists distinguished between adequate bowel preparation and HQC, with their interpretations aligning with current standards,^5,7,8,19^ where HQC corresponds with BBPS scores of 8–9 or a rating of ‘excellent’ on the Aronchick scale.^9,11^ In addition, the survey findings indicated that nearly all Italian endoscopists consider HQC to be essential across all clinical indications, with perceived beneficial impacts on ADRs, SSL detection, surveillance interval accuracy and overall colonoscopy efficiency.

Importantly, current guidelines endorse a performance target for endoscopy units of good bowel preparation (defined as a BBPS score of ⩾6, with each segment score ⩾2) in ⩾90% of all colonoscopies and ⩾95% of screening colonoscopies.^5,7,19^ In contrast, the current survey findings revealed that most endoscopists consider HQC as a priority in their clinical practice and experience; they want not only excellent preparation but also optimal bowel preparation (i.e. without the need for excessive washing) immediately prior to initiating colonoscopy, which extends beyond the concept of total BBPS scores of 8–9. Clearly, endoscopists in real-world clinical practice in Italy aim to achieve a standard of bowel preparation that exceeds key performance measures recommended by current guidelines.

HQC makes the endoscopist feel more relaxed about the results of the procedure, whereas suboptimal preparation reduces the endoscopist’s level of diagnostic confidence. This finding is important, as it highlights the psychological and operational value associated with bowel preparation, indicating a paradigm shift from ‘adequate’ to ‘high-quality’ cleansing.

Achieving high-quality bowel cleansing may also improve procedural efficiency by reducing the need for repeat colonoscopies and optimising the organisation of endoscopy services. Previous analyses have suggested that improved cleansing quality may contribute to more efficient use of healthcare resources.^32–35^

Importantly, although the literature cites clinical and economic advantages associated with HQC, several barriers exist including conceptual, organisational and economic considerations that prevent the achievement of HQC in clinical practice.^15–17,24,36–38^ For example, the lack of a standard definition for HQC and the fact that the quality of colonoscopy procedures and their interpretation is provider-dependent.^15–17^ A systematic literature review has identified a lack of consistent use of standardised measures of adequate bowel cleansing, with some studies using validated scales such as the BBPS or Aronchick scale, while many other studies have used unvalidated rating scales or they have not reported how bowel preparation was assessed.^32^ Introducing procedure standardisation, quality assurance standards and key performance indicators could reduce discrepancies in outcomes between different endoscopists, and potentially result in HQC.^24,36–38^

Efforts to improve the quality of bowel preparation and improve bowel cleansing outcomes include use of overnight split-dose or same-day regimen versus the day before dosing,^6,28,39–43^ tailored preparation protocols,^44^ delivery of enhanced education provided by trained healthcare professionals,^5^ patient navigation programmes,^45^ multimedia messaging services or educational programmes, including computer-based educational materials,^4,46–50^ a smart phone application to assess bowel preparation according to the appearance of the faeces in the toilet^51^ and the use of bowel preparation regimens designed to improve patient adherence, tolerability and cleansing success.^22,23,28,35,52–59^

Regarding lower-volume cleansing preparations, in addition to the aforementioned advantages, published evidence supports the use of lower-volume polyethylene glycol-based regimens in special populations such as hospitalised inpatients^58^ and the elderly.^60,61^ Additionally, published evidence suggests that such regimens can achieve HQC in the entire colon and/or the right colon (Table 3) and may represent one option among multiple guideline-supported regimens and implementation measures to facilitate achieving HQC. The choice of bowel preparation regimen in clinical practice should therefore be guided by patient characteristics, local organisational factors and current guideline recommendations.

**Table 3.** Selected evidence on bowel preparation regimens and high-quality cleansing outcomes.

Study	Study design	No. of pts	Comparator agent	Outcomes^a^
Bednarska 2022^57^	Qualitative, prospective, multi-centre, comparative, single-blind, observational study	1098	1L PEG + Asc vs 2L PEG + Asc	Adequate bowel cleansing (total BBPS score ⩾6 with a partial BBPS ⩾2 in each segment): 97% vs 95%, *p* = 0.100 HCQ (total BBPS score = 9): 73% vs 45%, *p* < 0.001 HQC right colon (BBPS right colon = 3): 77% vs 51%, *p* < 0.001
			1L PEG + Asc vs 4L PEG	Adequate bowel cleansing (total BBPS score ⩾6 with a partial BBPS ⩾2 in each segment): 97% vs 97%, *p* = 0.877 HCQ (total BBPS score = 9): 73% vs 53%, *p* < 0.001 HQC right colon (BBPS right colon = 3): 77% vs 58%, *p* < 0.001
Choi 2022^62^	Prospective, randomised, non-inferiority, open-label, phase 4 clinical trial	173	CleanViewAL® vs Plenvu® (both 1L PEG + Asc regimens)	Bowel cleansing success (total BBPS ⩾ 6 with a partial BBPS ⩾ 2 in each segment): 97.6% vs 98.8%, *p* = 0.207
Hong 2022^39^	Multi-centre, randomised, endoscopist-blinded, non-inferiority phase 4 study	360	1L PEG + Asc vs 2L PEG + Asc	Primary endpoint: Overall bowel cleansing success (BBPS score ⩾2 for all segments of the colon): 93.1% vs 91.9%, *p*_non-inferiority_ < 0.001 Secondary endpoints: (1) segmental bowel cleansing success rate (segmental colon BBPS score ⩾2): 95.4% vs 94.2%, *p*_non-inferiority_ < 0.001; (2) high-quality bowel cleansing (overall, BBPS score = 9, segmental colon, BBPS score = 3): 49.4% vs 37.8%, *p*_non-inferiority_ < 0.001; (3) Overall PDR: 48.9% vs 37.8%, *p*_non-inferiority_ < 0.001; (4) Overall ADR: 24.7% vs 20.4%, *p*_non-inferiority_ < 0.001
Lopez-Jamar 2023^6^	Observational, retrospective, multi-centre study	13,169	1L PEG + Asc overnight split-dose vs same-day regimen	Main endpoints: (1) overall adequate colon cleansing (BBPS score ⩾6 with a BBPS score ⩾2 in each segment): 94.7% vs 86.7%, *p* < 0.0001; (2) HQC in the right colon (BBPS score = 3): 65.4% vs 41.4%, *p* < 0.0001; (3) HQC in the total colon (BBPS score ⩾8): 72.0% vs 43.7%, *p* < 0.0001 Main exploratory endpoints: (1) PDRs in the total colon and right colon^b^: 48.9% vs 49.4%, *p* = 0.60 (2) ADR in the total colon and right colon^c^: 42.5% vs 24.3%^d^
Machlab 2025^60^	Post hoc analysis of an observational, multi-centre, retrospective study	423 (⩾80 years)	1L PEG + Asc	Main endpoints: (1) adequate bowel cleansing (BBPS score ⩾6 with a BBPS score ⩾2 in each segment): 88.9% (2) overall HCQ (BBPS score ⩾8 for the overall colon): 54.1% (3) HCQ in the right colon (BBPS score = 3 for the right colon): 46.1%
Maida 2023^41^	Meta-analysis of randomised controlled trials	–	1L PEG + Asc vs other bowel preparations	Cleansing success (BBPS score ⩾6 with a partial BBPS score ⩾2 in each segment): OR = 1.50; 95% CI 1.25, 1.81; *p* < 0.01 Right-colon HQC (partial BBPS score = 3): OR = 1.67; 95% CI 1.21, 2.31; *p* < 0.01 ADR (pooled estimate): OR = 1.02; 95% CI 0.87, 1.20; *p* = 0.79
Pérez Arellano 2024^63^	Randomised controlled trial	876	1L PEG + Asc vs SPMC	Primary endpoints: (1) Adequate bowel cleansing in the overall colon (BBPS score ⩾6 with a BBPS score ⩾2 in each segment): 90.8% vs 77.7%, *p* < 0.001 (2) Whole colon preparation quality: - excellent (total BBPS score 8–9): 55.2% vs 25.4%, *p* < 0.001 - good (total BBPS score 6–7): 25.6% vs 52.2%, *p* < 0.001 - poor (total BBPS score 1–5): 9.2% vs 22.3%, *p* < 0.001 (3) Adequate cleansing of the right colon (BBPS score ⩾2): 92.0% vs 82.7%, *p* < 0.001 (4) HQC of the right colon (BBPS score = 3): - excellent (total BBPS score 8–9): 58.7% vs 27.2%, *p* < 0.001 - good (total BBPS score 6–7): 33.2% vs 55.5%, *p* < 0.001 - poor (total BBPS score 1–5): 5.6% vs 15.9%, *p* < 0.001 Secondary endpoint: PDR in the overall colon^e^ 47.2% vs 42.2%, *p* = 0.139
Repici 2021^22^	Multicentre, single-blind, randomised controlled non-inferiority trial	388	1L PEG + Asc vs 4L PEG	Bowel preparation quality for entire colon (BBPS score ⩾6 with a BBPS score ⩾2 in each segment): 97.9% vs 93.0%, *p*_non-inferiority_ < 0.001 Bowel cleansing quality: - excellent/HQC (total BBPS score 8–9): 78.7% vs 64.5% - good (total BBPS score 6–7 and each colon segment score ⩾2): 18.6% vs 28.0% - fair (total BBPS score 3–5 or total BBPS score 6–7, but ⩾1 colon segment score < 2): 2.7% vs 6.5% - poor (total BBPS score 0–2): 0 vs 1.1%
Vassallo 2024^28^	Multicentre, single-blind, randomised controlled non-inferiority trial	478	1L PEG + Asc vs 4L PEG	Primary endpoint: overall cleansing success (total BBPS ⩾6, with a segmental BBPS ⩾2): 91.8% vs 83.6%, *p* = 0.009 Secondary endpoints: (1) excellent cleansing of the right colon (total BBPS score 9): 39.1% vs 27.7%, *p* = 0.001 (2) HQC of the right colon (total BBPS score = 3): 52.3% vs 38.5%, *p* = 0.004

This table is illustrative and non-exhaustive, is provided for contextual purposes, and does not imply endorsement of any specific product.

a Data are reported to one decimal place where known.

b PDRs in the total colon and right colon were defined as the proportion of colonoscopies, where ⩾1 polyp was found and removed.

c ADR in the total colon and right colon was defined as the proportion of colonoscopies, where ⩾1 adenoma was found as determined by histological analysis.

d The study reports the ADR only as overall values for the total colon and right colon, not for each dosing regimen.

e PDR in the overall colon was defined as the proportion of colonoscopies, where ⩾1 polyp was found and removed.

ADR, adenoma detection rate; Asc, ascorbate; BBPS, Boston Bowel Preparation Scale; CI, confidence interval; HCQ, high-quality cleansing; No., number; OR, odds ratio; PDR, polyp detection rate; PEG, polyethylene glycol; PEG + Asc, polyethylene glycol plus ascorbate; pts, patients; RR, relative risk; SPMC, sodium picosulphate with magnesium citrate.

### Limitations

Some limitations of this study should be acknowledged. First, selection bias may be present, as online surveys imply a certain degree of computer literacy and may exclude less digitally engaged respondents. Second, as an exploratory survey based on self-reported data, the findings may be affected by recall, response and social desirability bias and could not be independently verified against objective quality indicators or endoscopy reporting systems. Future studies combining survey findings with objective quality indicators (e.g. audit of endoscopy reports) would help validate and extend these results. Third, because the sampling strategy focused on experienced, high-volume, predominantly hospital-based endoscopists and excluded ‘opinion leaders’ as operationally defined above, the findings may not be fully generalisable to all endoscopists, particularly those working predominantly in private practice or those with high academic visibility. Finally, as this study was conducted in Italy, the findings may not be directly transferable to other healthcare systems where screening pathways, reimbursement models and endoscopy service organisation differ.

## Conclusion

The results of this survey reinforce the central role of high-quality bowel cleansing in colonoscopy and support the transition from ‘good’ to ‘high-quality’ cleansing as the new standard in clinical practice. HQC has demonstrated multiple benefits over adequate bowel preparation, including higher adenoma and SSL detection rates, a significant improvement in ADRs across all colonic segments, as well as a reduced risk of PCCRC. Other advantages include an improved procedural efficiency and better overall clinical outcomes. Future research should focus on standardising HQC criteria, improving patient adherence through enhanced education strategies and evaluating the long-term cost-effectiveness of adopting HQC as the default standard in colonoscopy procedures. By prioritising HQC, the gastroenterology community can ensure more effective CRC prevention, improved diagnostic accuracy and optimised endoscopic efficiency, ultimately enhancing patient outcomes.

## Supplemental Material

sj-docx-1-cmg-10.1177_26317745261441726 – Supplemental material for Toward high-quality bowel preparation in Italy: insights from a nationwide cross-sectional survey of endoscopists Supplemental material, sj-docx-1-cmg-10.1177_26317745261441726 for Toward high-quality bowel preparation in Italy: insights from a nationwide cross-sectional survey of endoscopists by Cesare Hassan, Roberto Di Mitri, Lorenzo Fuccio, Marcello Maida, Gianpiero Manes, Mauro Manno, Franco Radaelli, Cristiano Spada, Roberto Vassallo and Alessandro Repici in Therapeutic Advances in Gastrointestinal Endoscopy

sj-docx-2-cmg-10.1177_26317745261441726 – Supplemental material for Toward high-quality bowel preparation in Italy: insights from a nationwide cross-sectional survey of endoscopists Supplemental material, sj-docx-2-cmg-10.1177_26317745261441726 for Toward high-quality bowel preparation in Italy: insights from a nationwide cross-sectional survey of endoscopists by Cesare Hassan, Roberto Di Mitri, Lorenzo Fuccio, Marcello Maida, Gianpiero Manes, Mauro Manno, Franco Radaelli, Cristiano Spada, Roberto Vassallo and Alessandro Repici in Therapeutic Advances in Gastrointestinal Endoscopy

## Declarations

**Ethics approval and consent to participate:** Ethics approval was not required for this survey. Consent to participate was implicit.

**Consent for publication:** Not applicable.

**Availability of data and materials:** The datasets used and/or analysed during the current study are available from the corresponding author on reasonable request.
