# Supplementary material for: Toward high-quality bowel preparation in Italy: insights from a nationwide cross-sectional survey of endoscopists
Source: Ther Adv Gastrointest Endosc. 2026 May 11;19:26317745261441726. doi: 10.1177/26317745261441726 (PMC13167322; doi:10.1177/26317745261441726)
Supplement: sj-docx-1-cmg-10.1177_26317745261441726 – Supplemental material for Toward high-quality bowel preparation in Italy: insights from a nationwide cross-sectional survey of endoscopists [file sj-docx-1-cmg-10.1177_26317745261441726.docx]

**Toward High-Quality Bowel Preparation in Italy: Insights from a Nationwide Cross-Sectional Survey of Endoscopists**

**Authors:** Cesare Hassan, Roberto Di Mitri, Lorenzo Fuccio, Marcello Maida, Gianpiero Manes, Mauro Manno, Franco Radaelli, Cristiano Spada, Roberto Vassallo, Alessandro Repici

**Supplementary material**

**Survey questions and possible answers**

1. In which region do you practice the profession of gastroenterologist endoscopist?

- Northwest
- Northeast
- Centre
- South and Islands

2. Where do you mainly carry out your activity as a gastroenterologist endoscopist?

- Public hospital
- Private accredited hospital
- Private hospital
- Local Health Authority clinic

3. In a year, approximately, how many colonoscopies do you personally perform?

- 200−499
- 500−549
- 550−599
- ≥600

4. Which is your age (completed years)?

- ≤45
- 46−55
- 56−60
- 61−65

5. How many years have you been practicing as an endoscopist gastroenterologist, without considering the years of specialization and internship?

- 5−15
- 16−20
- 21−25
- > 25

6. Do you routinely (i.e., in most exams) evaluate and report the quality of the bowel preparation in the endoscopic report?

- I use validated scales
- I don’t use validated scales

7. In your clinical practice, what is the cleansing goal you aim for with a bowel preparation?

- Get an optimal cleansing (high quality)
- Get a good cleansing
- Obtain sufficient cleansing to answer the clinical question

8. Based on your experience, how would you define good bowel cleansing? From the following, choose the definition that best describes adequate bowel cleansing for you.

- A cleansing that meets criteria validated by the preparation scales (e.g. score >=2 according to the Boston Bowel Preparation Scale; at least fair preparation – “fair” according to the Aronchick scale)
- A cleansing that makes the mucosa visible without excessive washing and suction
- A cleansing that satisfies the endoscopist in making a confident diagnosis in relation to the clinical question of the examination

9. Based on your experience, how would you define optimal (high-quality) bowel cleansing? From the following, choose the definition that best describes adequate bowel cleansing for you.

- A cleansing with high scores (e.g. score of 8-9 according to the Boston Bowel Preparation Scale; excellent according to the Aronchick scale)
- A cleansing that allows optimal visualization of the mucosa without the need for washing and aspiration during the examination
- A cleansing that satisfies the endoscopist in making a confident diagnosis in relation to the clinical question of the examination

10. Do you think that optimal (high-quality) intestinal cleansing …?

- Be associated with a higher rate of adenoma identification than a sufficient preparation alone
- Be associated with a higher rate of identification of serrated sessile lesions compared to only sufficient preparation
- Reduce examination times and improve endoscopy efficiency
- It may be associated with better appropriateness of the surveillance intervals

11. Do you think that achieving optimal (high-quality) bowel cleansing is important …?

- In every colonoscopy
- Mainly for certain indications (e.g. a positive FIT result for screening colonoscopy)

12. Could you please indicate your state of mind, how confident you feel after having a colonoscopy with a cleansing …? In answering, please use a scale from 1 to 10, where 1 corresponds to “it doesn’t make me feel confident at all” and 10 corresponds to “it makes me feel very confident”.

- Inadequate
- Good
- High Quality
